# Arthroscopic-Assisted Pediatric Transitional Ankle Fracture Reduction and Fixation Technique

**DOI:** 10.7759/cureus.94919

**Published:** 2025-10-19

**Authors:** Eunice Anastasia Wilianto, Neeraj Mishra, Derrick Jun Liang Lam, Andrew Chia Chen Chou, Kenneth Pak Leung Wong, Mohammad Ashik Bin Zainuddin

**Affiliations:** 1 Yong Loo Lin School of Medicine, National University of Singapore, Singapore, SGP; 2 Orthopaedic Surgery, KK Women's and Children's Hospital, Singapore, SGP

**Keywords:** arthroscopic reduction internal fixation, arthroscopy, new surgical technique, orthopedic, pediatric fractures, pediatric orthopedic surgery, surgical technique, tillaux fracture, transitional ankle fracture, triplane fracture

## Abstract

This paper describes and evaluates an arthroscopic technique for arthroscopic reduction and fixation (ARIF) of transitional ankle fractures, presenting its preliminary clinical outcomes.

This is a retrospective technical report on prospectively collected data from patients who underwent ARIF of transitional ankle fractures. Demographic information, along with clinical details such as injury type, duration of surgery, length of hospital stay, and maximum Visual Analog Scale (VAS) pain scores, were prospectively collected. Postoperatively, patients were followed up for a minimum of one year, where complications, time to achieve full weight bearing, and radiological evidence of fracture healing were all documented. Functional outcomes were also evaluated using the average Foot and Ankle Outcome Score (FAOS) and the American Academy of Orthopedic Surgeons (AAOS) score at six-month follow-up. Clinical outcomes were assessed to evaluate the effectiveness of this ARIF technique.

Three patients, two (67%) females and one (33%) male, with a mean age of 13 ± 1 years, underwent the arthroscopically assisted fixation method for low-energy fractures, including two (67%) Tillaux fractures and one (33%) two-part triplane fracture. The average duration of surgery was 93 ± 7 minutes. Postoperatively, the maximum VAS pain score recorded was 4 ± 1 during hospitalization, and all patients were discharged the following day. The average time to full weight-bearing was 45 ± 6 days, while radiological healing occurred on average at 57 ± 7 days. At the six-month follow-up, the average FAOS was 92 ± 5, and the AAOS score was 93 ± 3. Functional outcomes were excellent and may surpass those of previously reported ARIF techniques.

In conclusion, the arthroscopically assisted fixation technique described in this study for Tillaux and two-part triplane fractures has yielded outstanding clinical outcomes, demonstrating excellent healing and functional recovery. By offering a safer, more controlled means of achieving precise fracture reduction, it enhances surgical efficiency without compromising workflow. Moreover, its reproducibility and reliance on standard surgical instruments make it a practical and accessible option for widespread adoption across varied clinical settings.

## Introduction

Ankle fractures constitute approximately 5% of all pediatric fractures and represent 9% to 18% of physeal injuries in children [1,2]. Transitional ankle fractures include Tillaux and triplane fractures, which account for approximately 3-5% and 5-15% of pediatric ankle fractures, respectively [1,3]. These transitional ankle fractures are distinctive injuries that occur exclusively during mid-adolescence, a period marked by the gradual and uneven closure of the distal tibial physis. This physeal closure begins centrally, proceeds medially, and ultimately concludes anterolaterally. This unique, asymmetrical pattern of physeal fusion gives rise to the characteristic fracture patterns seen in transitional ankle fractures [4]. These fractures bridge the transition between the skeletally immature and mature ankles and reflect the changing biomechanical properties of the growth plate during this developmental window. Managing these transitional fractures presents a significant challenge due to their intra-articular location within the confined ankle joint, necessitating meticulous reduction and stable fixation to restore joint congruity and prevent long-term complications.

Intra-articular fractures exhibiting more than 2 mm of displacement accompanied by disruption of the distal tibial articular surface mandate reduction under anesthesia. The current standard of care involves open reduction and internal fixation (ORIF), typically utilizing wires or screws for stabilization [5,6]. Alternatively, closed reduction and percutaneous pinning (CRPP) may be employed, using Kirschner wires as a joystick to achieve precise reduction. While CRPP offers the benefits of minimal scarring, reduced surgical trauma, and faster recovery, its application is limited. Soft tissue interposition frequently impedes adequate closed reduction, restricting its use to minimally displaced or nondisplaced fractures that are at risk of future instability.

In recent years, arthroscopy has gained increasing popularity in the management of ankle injuries, owing to its minimally invasive nature, expedited recovery, and reduced risk of surgical trauma [3]. Arthroscopic-assisted reduction and internal fixation (ARIF) is now a well-established modality in adult ankle fracture management; however, its adoption in the pediatric population remains limited and less clearly defined [2]. ARIF combines the benefits of CRPP, including minimal scarring, reduced soft tissue dissection, and faster rehabilitation, with the precision and stability of ORIF, offering the potential for superior anatomical reduction and clinical outcomes. Despite growing interest in minimally invasive approaches, there remains a paucity of robust evidence in the pediatric orthopedic literature regarding arthroscopically assisted techniques for the reduction and fixation of transitional ankle fractures [2,3,5,6].

In this article, we describe a novel arthroscopic technique for the reduction and fixation of transitional ankle fractures, using simple instruments readily available in an arthroscopy operating theater, for intra-articular reduction, and present its preliminary clinical outcomes to highlight its feasibility, safety, and potential advantages over existing methods.

This article was presented at the EFORT Congress 2024 in Hamburg and the APSS-APPOS-MSS Conference 2025 in Kuala Lumpur.

## Technical report

Description of fractures

**Figure 1:**
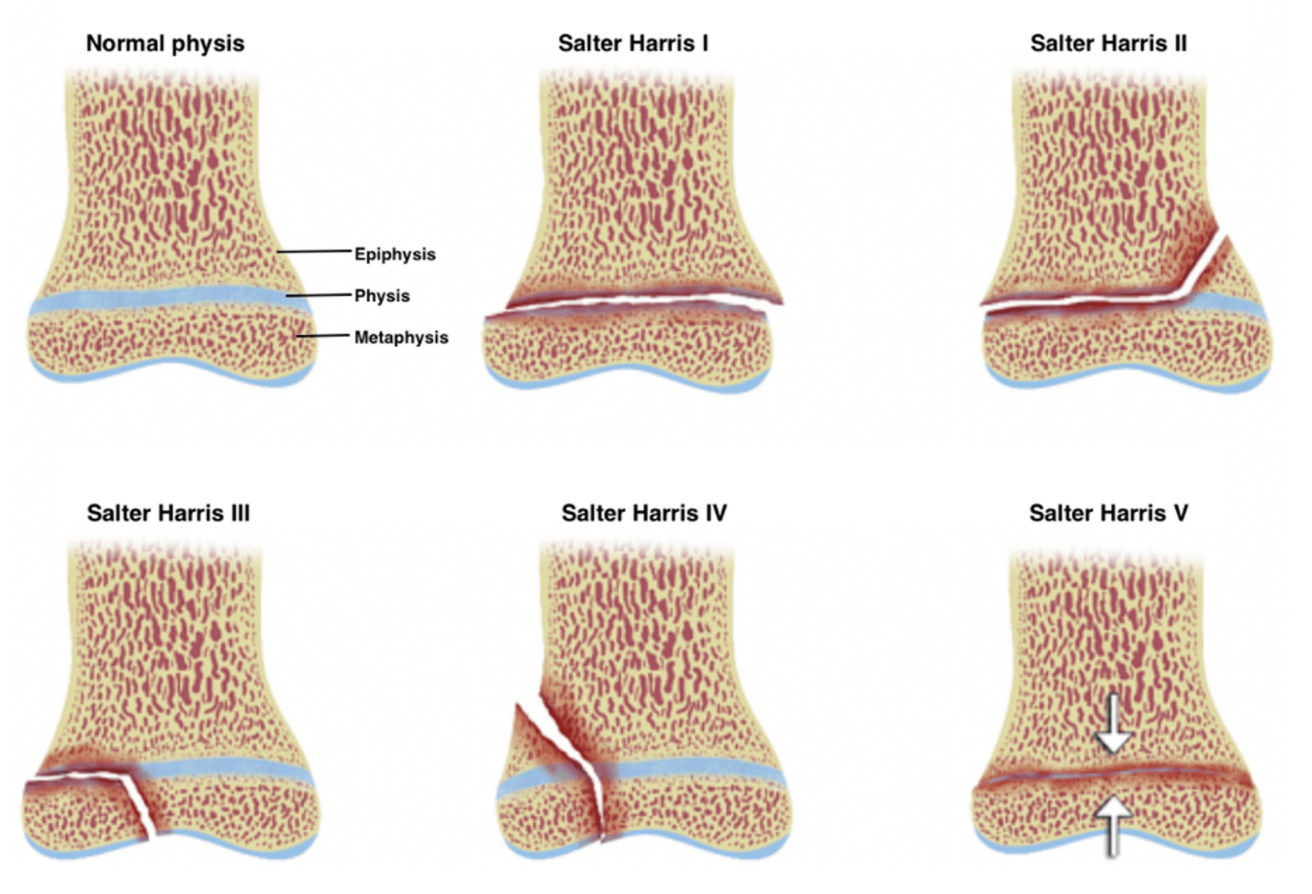
Salter-Harris fracture classification. Illustration depicting normal physis with intact epiphysis and metaphysis; type I fracture, extending through the physis; type II, with the fracture extending through physis and metaphysis; type III, with the fracture extending through the physis and epiphysis; type IV, with the fracture extending through the metaphysis, physis, and epiphysis; type V, with crushed/compression injury of the whole physis. Source: Figure adapted from Skalski M [7] under the Creative Commons Attribution-Noncommercial-Share Alike 3.0 Unported licence.

**Figure 2:**
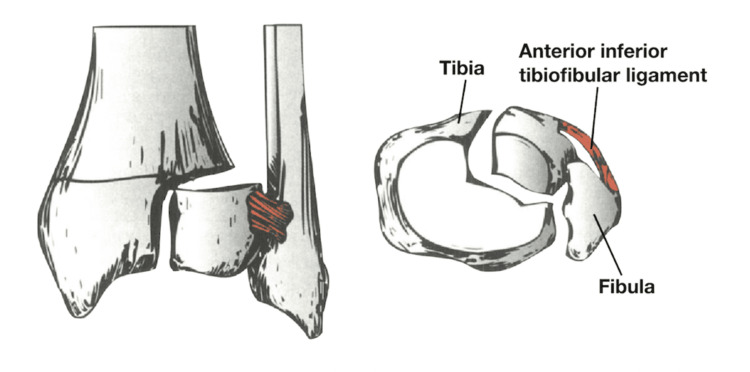
Tillaux fracture. Tillaux fracture, a Salter-Harris III fracture of the anterolateral distal tibia epiphysis. Source: Figure reproduced from Cadogan M and Gomez A [8] under the Creative Commons Attribution-NonCommercial-ShareAlike 4.0 International License.

**Figure 3:**
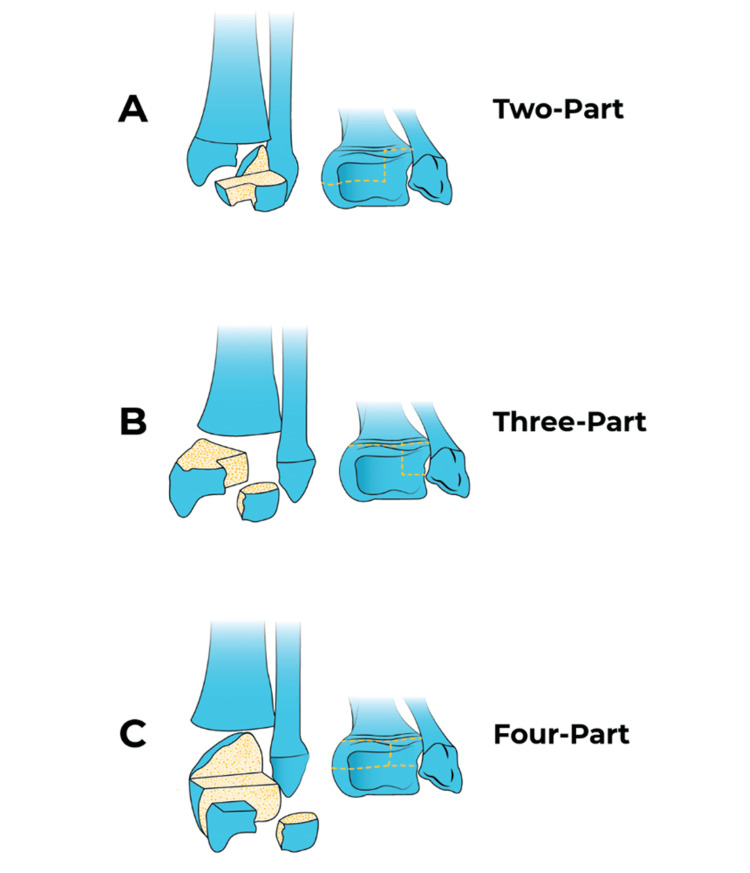
Triplane fractures. Triplane fractures, classified as in two, three, or four parts. Source: Figure reproduced from O'Leary A [9] under the Creative Commons Attribution License.

A Tillaux fracture is a Salter-Harris type III injury affecting the anterolateral distal tibial epiphysis, while a triplane fracture is a Salter-Harris type IV injury that extends through the epiphysis, physis, and metaphysis (Figures 1-3) [4,7-9].

Study design

This study reports a retrospective technical report based on prospectively collected data from patients who underwent arthroscopic-assisted reduction and fixation of transitional ankle fractures between October 2021 and May 2023. All procedures were performed by a single fellowship-trained senior pediatric orthopedic surgeon at a tertiary care center in Singapore.

Inclusion criteria comprised the following: (1) isolated closed Tillaux fractures, (2) isolated closed two-part triplane fractures, and (3) a minimum follow-up duration of one year. Patients with associated injuries, open wounds, other concurrent fractures, or three- and four-part triplane fractures were excluded from the study. Complex triplane fractures were excluded due to their fragment complexity and the likely need for open reduction, which we felt would not be safely or effectively addressed with the described arthroscopic method. All patients in our series underwent standard anteroposterior and lateral ankle X-rays initially, followed by a preoperative CT scan of the ankle. A single dose of intravenous first-generation cephalosporin was given preoperatively for antibiotic prophylaxis, as per standard protocol. Postoperative follow-up evaluations were conducted at one, two, four, and six months, as well as at the one-year mark.

Demographic information, along with clinical details such as injury type, affected side, duration of surgery, length of hospital stay, and maximum Visual Analog Scale (VAS) pain scores to assess pain intensity, were documented at the time of admission for surgery [10]. Postoperative follow-up assessments included documentation of complications, time to achieve full weight-bearing, and radiological evidence of fracture healing. Functional outcomes were evaluated at the six-month follow-up using the American Academy of Orthopedic Surgeons (AAOS) Foot and Ankle Questionnaire and the Foot and Ankle Outcome Score (FAOS) [11,12]. VAS, AAOS, and FAOS scoring tools are publicly available, validated tools that are freely accessible for clinical and research use. All patients were monitored for a minimum of one year.

Indications

**Figure 4:**
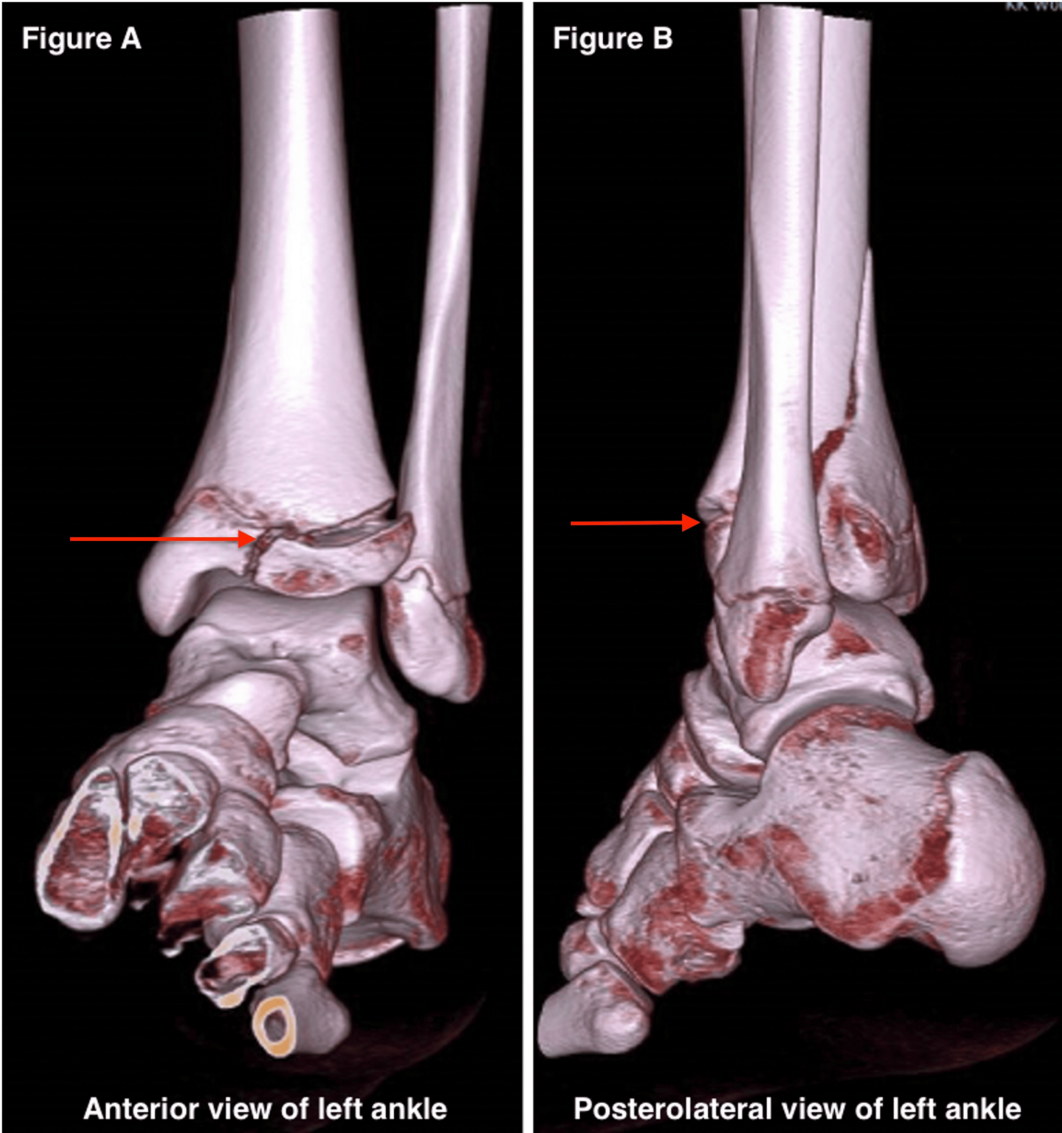
Preoperative computed tomography (CT) scan of a triplane fracture showing anterior (A, left) and posterolateral (B, right) views of the left foot. Arrows: Triplane fracture, a Salter-Harris type IV fracture at the distal tibia of the left ankle, involving metaphysis, physis (growth plate), and epiphysis.

This arthroscopically assisted technique is indicated for the management of pediatric Tillaux and two-part triplane ankle fractures. Figure 4 presents the preoperative CT scan of a triplane fracture of the left distal tibia.

Equipment and setup

The procedure requires a standard arthroscopy setup, including an arthroscopic pump and instruments, a large, pointed reduction clamp, a 12G needle, a 3.5 mm arthroscopic shaver, an image intensifier, 1.6 mm Kirschner wires, and a 4.0 mm short-thread cannulated screw.

Surgical technique

Patient Positioning

**Figure 5:**
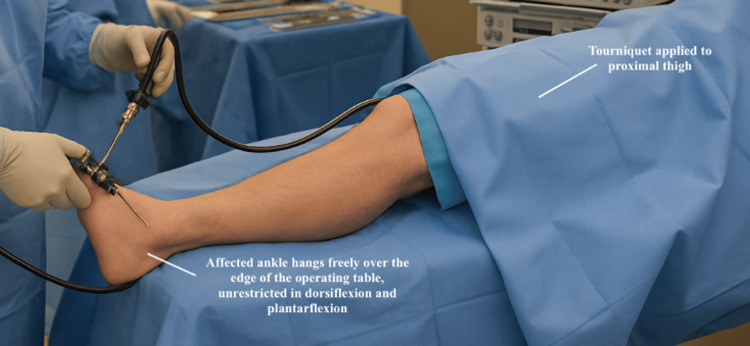
Patient positioning for ankle arthroscopy.

The patient is positioned supine with a tourniquet applied to the proximal thigh and appropriate padding over all bony prominences. The affected ankle is allowed to hang freely over the edge of the operating table, facilitating unrestricted dorsiflexion and plantarflexion during arthroscopy. Patient position is depicted in Figure 5.

Portal Placement

**Figure 6:**
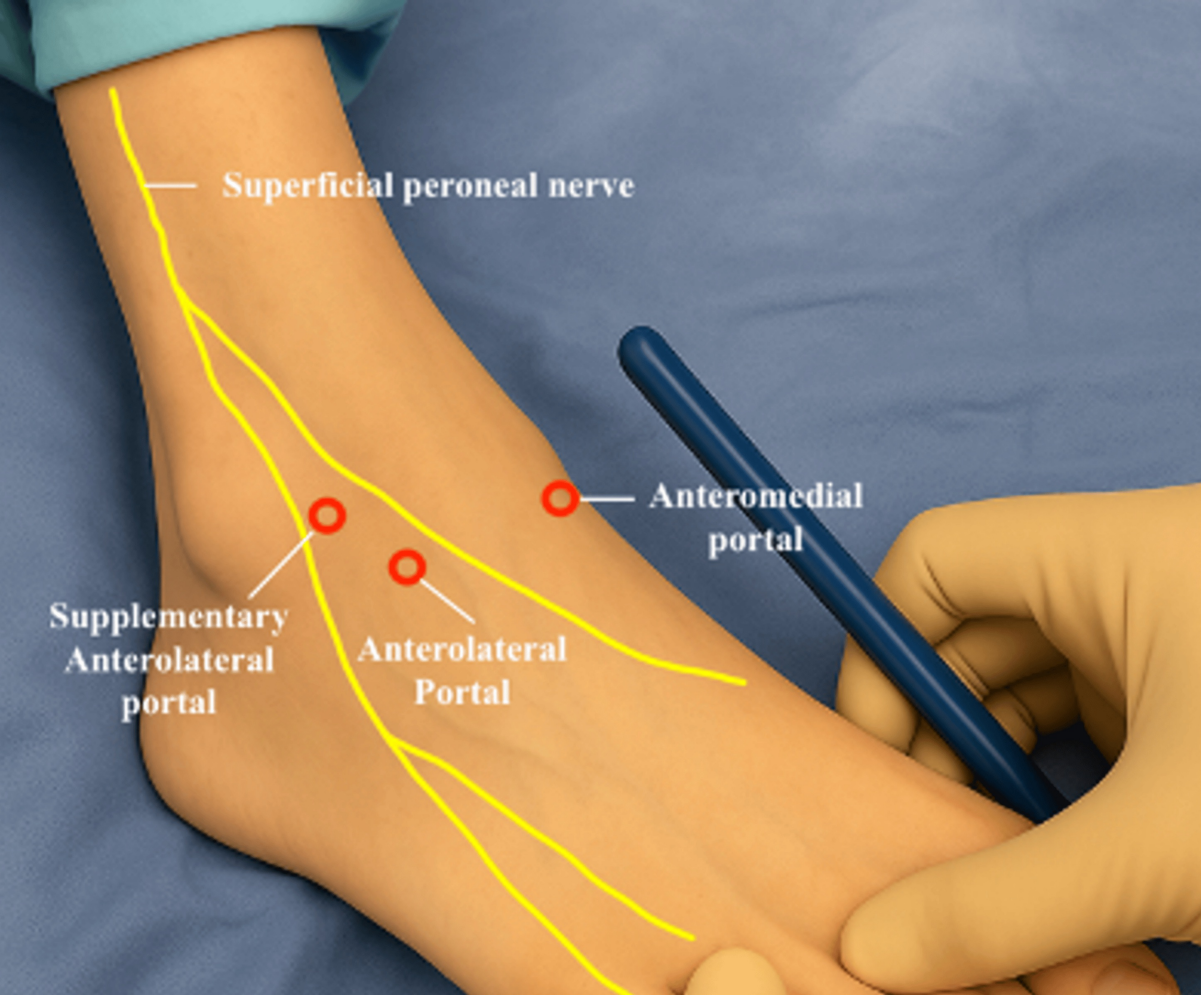
Portal placement for ankle arthroscopy showing the supplementary anterolateral, anterolateral, and anteromedial portals with respect to the superficial peroneal nerve.

The standard anteromedial and anterolateral portals are identified and marked with care taken to avoid branches of the superficial peroneal nerve, as shown in Figure 6.

Joint Insufflation and Scope Introduction

A 12G needle is inserted into the anteromedial portal to insufflate the ankle joint with normal saline, facilitating joint distension. A small skin incision is then made at the same site, through which the arthroscope is introduced. The anterolateral portal is subsequently established in a similar manner, ensuring appropriate triangulation for optimal visualization and instrumentation.

Debridement and Visualization

**Figure 7:**
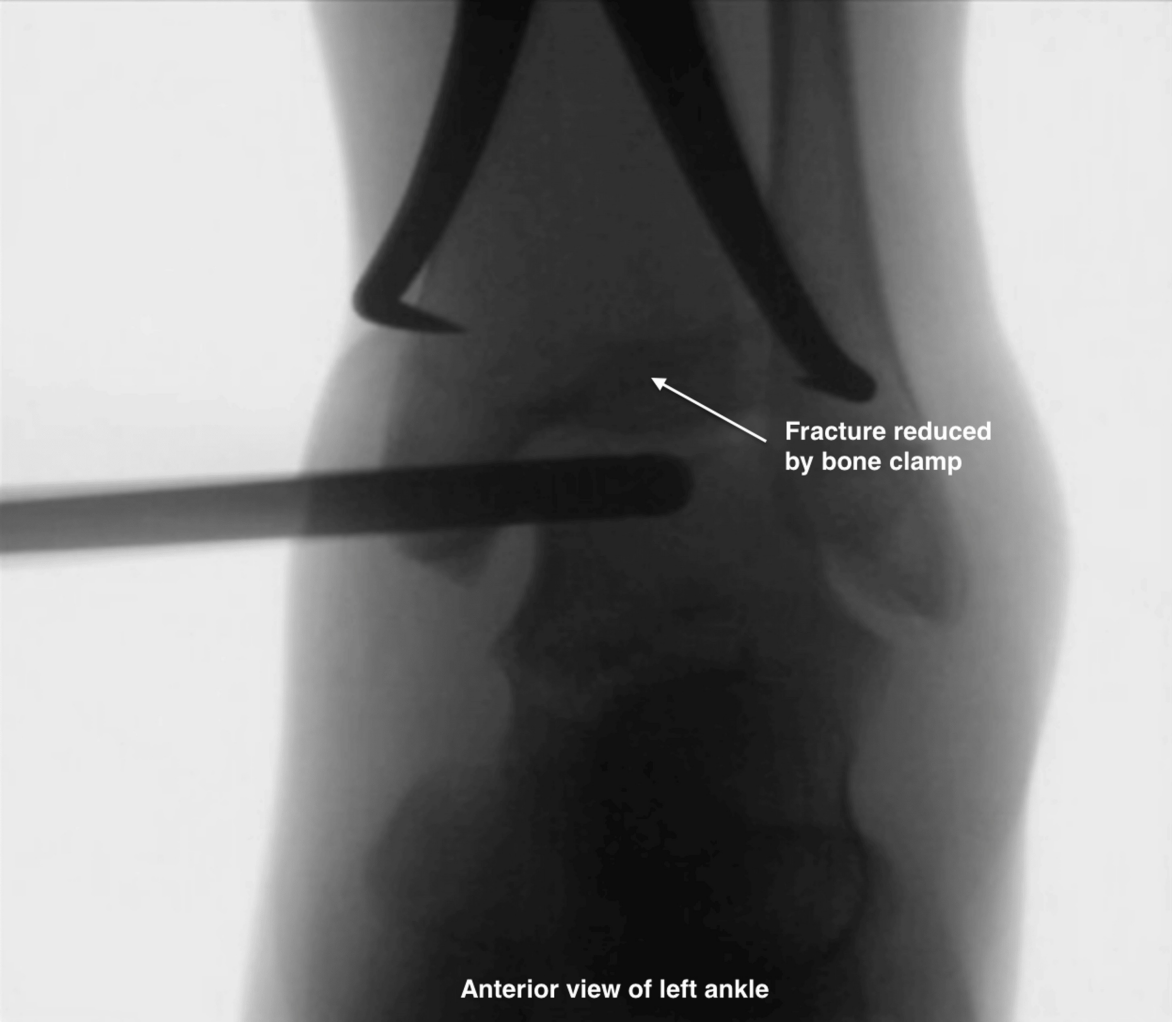
Fluoroscopic image of the fracture being reduced by a bone clamp; anterior view of the left ankle. Arrow: Triplane fracture fragments at the distal tibia are clamped and reduced by the bone clamp.

A 3.5 mm arthroscopic shaver is introduced through the anterolateral portal to perform thorough joint debridement, enhancing visualization of intra-articular structures, including the origin of the anterior inferior tibiofibular ligament (AITFL). Figure 7 presents the fluoroscopic image showing an arthroscope used for direct visualization of the fracture site and the fracture reduced by a bone clamp.

**Figure 8:**
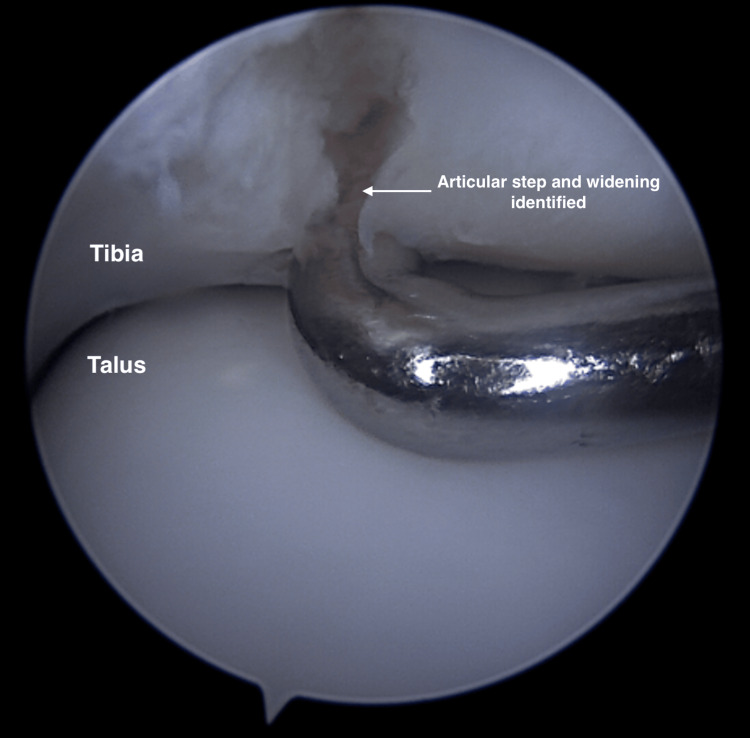
Articular step and widening identified. Fracture fragments are mobilized using the MacDonald retractor, and any interposing hematoma, fibrin, or soft tissue is cleared. Arrow: Articular step and widening at the distal tibia.

The fracture line is carefully identified, as shown in Figure 8, and cleared of hematoma, fibrin, and any interposing soft tissue debris. A MacDonald retractor is then inserted via the anterolateral portal to aid in the disengagement and mobilization of the fracture fragments, facilitating anatomic reduction.

AITFL Localization and Clamp Reduction

An 18G needle is used to localize the supplementary anterolateral portal, which is positioned approximately 1.0-1.5 cm lateral and 1.0-1.5 cm proximal to the standard anterolateral portal, ensuring direct access to the origin of the AITFL. A small skin incision is made, followed by blunt dissection to expose the AITFL origin. The pointed tip of the reduction clamp is introduced through the accessory portal and placed directly on the bony AITFL origin to enable precise fracture reduction while ensuring no soft tissue or cartilage is injured.

Fracture Fixation

**Figure 9:**
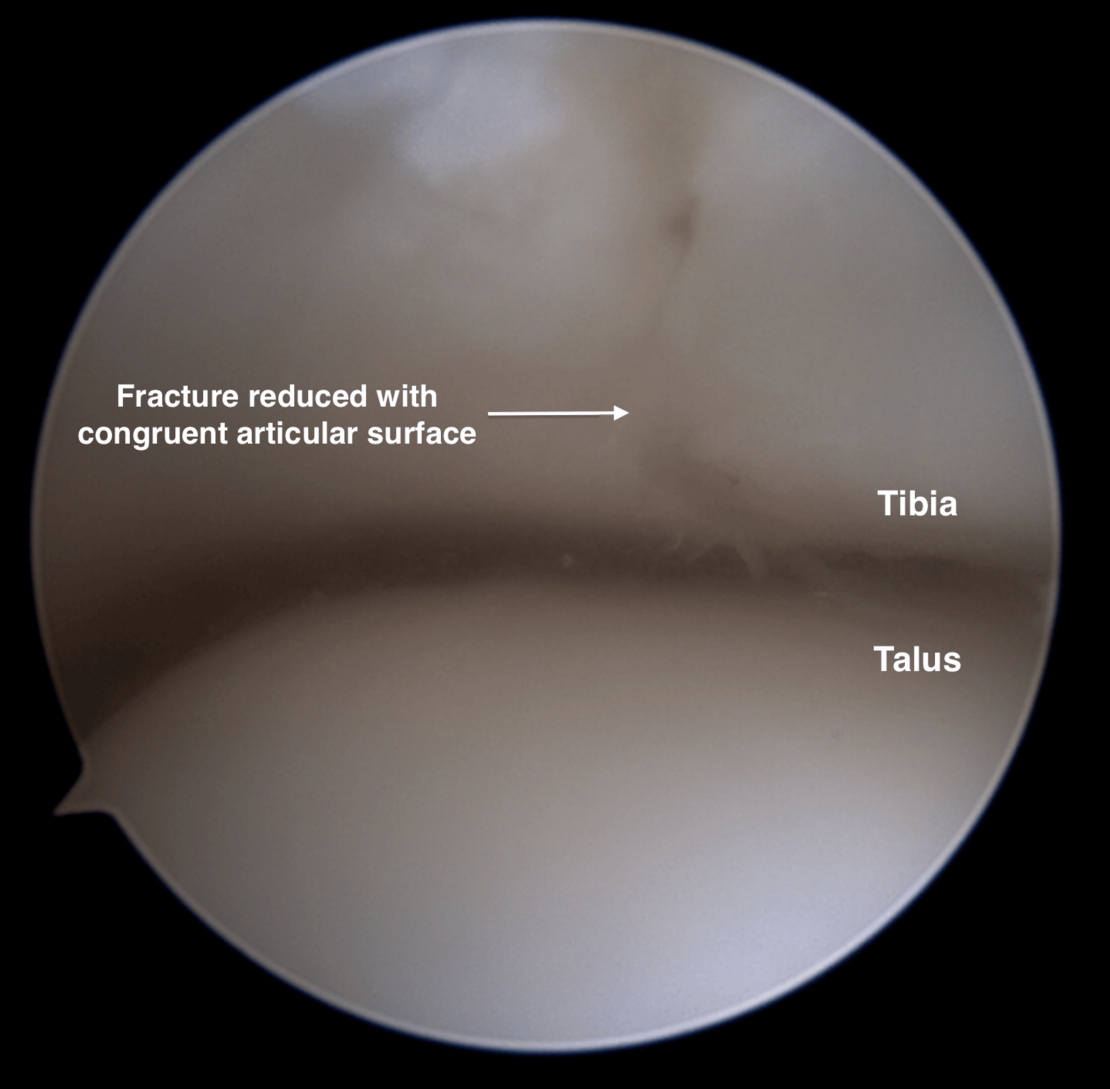
Fracture reduced with congruent articular surface. Arrow: Post-reduction view of the distal tibia. Fracture fragments are apposed, and the articular surface is congruent. No articular step or widening is seen.

Once the fracture reduction is achieved and confirmed both arthroscopically and fluoroscopically, as shown in Figure 9, a 1.6 mm Kirschner wire is inserted across the fracture site in a medial-to-lateral direction, starting from the medial malleolus area. A 4.0 mm short-thread cannulated screw is then passed over the wire, ensuring that all screw threads extend beyond the lateral aspect of the fracture line to achieve compression. Finally, the reduction integrity and stability are reassessed and confirmed using both arthroscopy and fluoroscopy.

Closure

The skin is closed using Prolene sutures, ensuring optimal wound closure and minimal tension.

Postoperative Management

**Figure 10:**
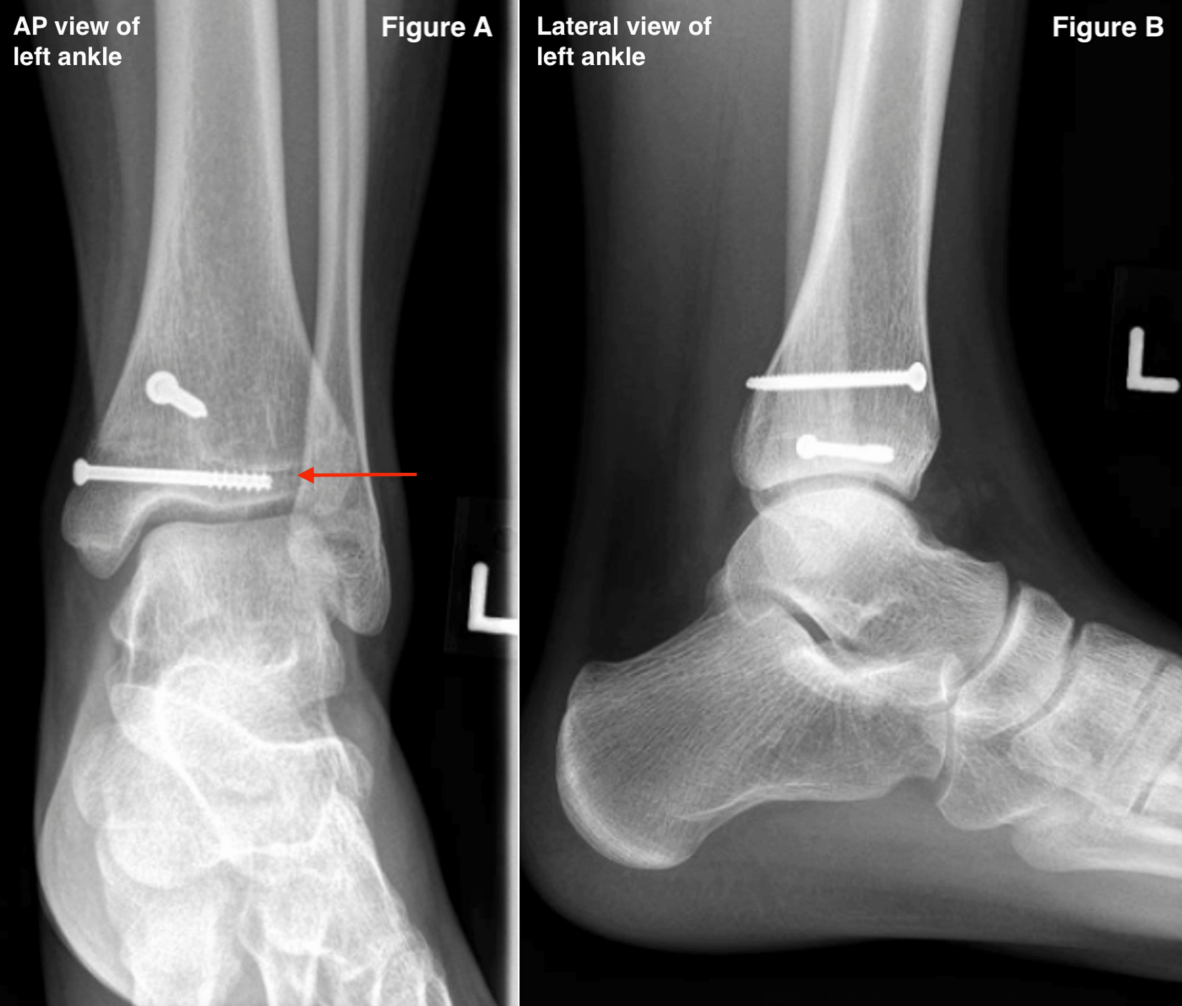
Postoperative radiographs showing healed fracture, with anteroposterior (A, left) and lateral views (B, right) of the left ankle. Arrow: Fracture fragments are held together by 4.0 mm short-thread cannulated screws for healing to occur. AP: anteroposterior.

Patients are instructed to wear a walker boot and remain non-weight bearing for the first four weeks. After this period, they progress to partial weight-bearing for an additional four weeks. Full weight-bearing is permitted once eight weeks postoperatively [6]. Postoperative radiographs are taken to monitor healing progress during follow-ups, as seen in Figure 10.

Results

Three patients, two (67%) females and one (33%) male, with a mean age of 13 ± 1 years, underwent the arthroscopically assisted fixation method for two (67%) Tillaux fractures and one (33%) two-part triplane fracture. Ethnically, two (67%) patients were of Chinese descent, and one (33%) was Malay. All fractures resulted from low-energy trauma. The average duration of surgery was 93 ± 7 minutes. Postoperatively, the maximum VAS pain score recorded was 4 ± 1 during hospitalization, and all patients were discharged the following day [10]. The average time to full weight-bearing was 45 ± 6 days, while radiological healing occurred on average at 57 ± 7 days. At the six-month follow-up, the average FAOS was 92 ± 5, and the AAOS score was 93 ± 3. No complications were observed in any of the patients (Tables 1, 2) [11,12].

**Table 1:** Demographics of patient characteristics and clinical outcomes for arthroscopically assisted fixation of Tillaux and two-part triplane fractures. FAOS: Foot and Ankle Outcome Score; AAOS: American Academy of Orthopedic Surgeons.

Parameters	Transitional ankle fractures (n = 3)
Age, years (mean ± SD)	12.9 ± 0.6
Sex, male, n (%)	1 (33.3%)
Sex, female, n (%)	2 (66.7%)
Race, Chinese, n (%)	2 (66.7%)
Race, Malay, n (%)	1 (33.3%)
Side of injury, left, n (%)	2 (66.7%)
Side of injury, right, n (%)	1 (33.3%)
Tillaux fractures, n	2 (66.7%)
Two-part triplane fractures, n	1 (33.3%)
Duration of surgery, minutes (mean ± SD)	92.7 ± 6.9
Length of stay, days (mean ± SD)	1.0 ± 0.0
Maximum visual analog pain score recorded during admission (mean ± SD)	4.3 ± 1.2
Time to full weight bearing, days (mean ± SD)	45.0 ± 5.7
Time to full radiological healing, days (mean ± SD)	56.7 ± 6.8
FAOS scores at 6 months (mean ± SD)	91.7 ± 4.7
AAOS foot and ankle outcome questionnaire scores at 6 months (mean ± SD)	92.7 ± 2.5

**Table 2:** Patient information and clinical outcomes. VAS: Visual Analog Scale; FAOS: Foot and Ankle Outcome Score; AAOS: American Academy of Orthopedic Surgeons.

Patient number	Age (years, months)	Sex, race	Type of fracture, side	Duration of surgery, minutes	Length of hospital stay, days	Max. VAS recorded	Time to full weight bearing, days	Time to full radiological healing, days	FAOS score at 6 months follow-up	AAOS foot and ankle outcome questionnaire scores at 6 months follow-up	Complications
1	12 years, 2 months	F, Chinese	Triplane, left	101	1	6	52	66	95	92	Nil
2	13 years, 3 months	F, Malay	Tillaux, right	84	1	4	45	54	85	90	Nil
3	13 years, 6 months	M, Chinese	Tillaux, left	93	1	3	38	50	95	96	Nil

## Discussion

Tillaux fractures are intra-articular fractures of the distal tibia, caused by avulsion of the anterolateral portion of the distal tibial epiphysis at the site of attachment of the AITFL (Figure 2) [5]. Triplane fractures, on the other hand, are injuries of the distal tibia that can involve two, three, or even four main fragments, depending on the mechanism of injury and the maturity of the physeal plate (Figure 3) [13,14]. In this study, we included two Tillaux fractures and one two-part triplane fracture, and we describe a novel arthroscopic technique for reducing and fixing transitional ankle fractures. Using straightforward instruments commonly found in an arthroscopy theater, the method enables precise intra-articular reduction. We also present preliminary clinical outcomes to demonstrate the technique’s feasibility, safety, and potential advantages compared with existing approaches.

The first case of arthroscopically assisted reduction and fixation of a Tillaux fracture was reported in 2002 by Leetun et al. [15], while the earliest documented use of arthroscopic treatment for triplane fractures dates back to 1993 by Whipple et al. [16], involving two distal tibial triplane fractures. These early reports highlighted the advantages of arthroscopic observation, which allowed for superior visualization of articular surfaces, ensured precise reduction, and minimized the risk of pin and screw placement across the joint surface or physis, thus preventing potential complications such as growth arrest, degenerative arthritis, cartilage damage, and joint stiffness [15,16]. Since these initial reports, the technique has steadily gained popularity among both surgeons and patients due to its numerous benefits. It allows for more accurate and less traumatic reduction compared to traditional CRPP and ORIF, respectively [15]. It is well established that patients who undergo arthroscopic-assisted procedures typically experience quicker recovery, with a lower incidence of stiffness and future osteoarthritic changes [17].

Transitional ankle fractures, occurring during the mid-adolescent phase of physeal maturation, often require precise management to optimize outcomes and minimize complications. Both CRPP and ORIF have been established as effective surgical interventions. Sojib et al. reported that CRPP offers several notable advantages over ORIF, including the avoidance of surgical incisions, which contributes to a lower risk of infection, reduced soft tissue trauma, improved cosmetic outcomes, and expedited recovery. In contrast, while ORIF allows for direct visualization and manipulation of fracture fragments, it is inherently more invasive and carries an increased risk of surgical complications [18]. Nevertheless, Asad et al. concluded that both techniques remain viable for the treatment of distal tibial physeal fractures, with CRPP demonstrating additional benefits in terms of reduced procedure-related morbidity [19].

Recent advancements have highlighted the value of arthroscopically assisted fixation as a superior, minimally invasive alternative that bridges the benefits of both CRPP and ORIF. Arthroscopy allows for direct visualization of the intra-articular surface and fracture configuration, enabling precise assessment and controlled reduction under real-time guidance. This approach significantly reduces the risk of joint incongruity and post-traumatic arthritis [20]. Compared to traditional open surgery, arthroscopic fixation is more efficient and accurate, with fewer complications, such as infection, soft tissue disruption, cartilage injury, and excessive bleeding [18]. It also preserves essential biological factors, such as native vascular supply, which are critical for optimal healing [21]. Furthermore, the minimally invasive nature of arthroscopy offers aesthetic advantages and facilitates faster postoperative recovery, benefits that are particularly valued by both patients and their families [22].

While arthroscopic surgery for Tillaux fractures is widely regarded as a promising method, the literature often lacks detailed descriptions of specific reduction techniques. Available studies reveal that different arthroscopic approaches have been utilized to achieve satisfactory reduction, which can be broadly classified into intra-articular [23], extra-articular [22,24], or a combination of both methods. In the intra-articular reduction technique, instruments are introduced through arthroscopic portals into the joint space to facilitate debridement, disengagement, and precise manipulation of the fracture fragments, enabling optimal anatomical reduction. In contrast, the extra-articular reduction technique relies on external manipulation, such as the use of clamps, Kirschner wires, or other instruments, while the reduction is monitored arthroscopically to ensure proper alignment. Although various reduction techniques have been explored, no comparative studies have yet evaluated the relative efficacy of intra-articular versus extra-articular methods.

The patients presented in this study utilized an intra-articular reduction technique, with the aid of a MacDonald's retractor, to disengage and reduce the fracture fragment. The MacDonald's retractor, known for its gentleness on the articular surface, offers a more protective approach for articular cartilage, compared to other instruments commonly used in fracture reduction. An alternative intra-articular reduction method, as reported by Fonseca et al. in a Tillaux fracture case, involves using a blunt instrument, such as the posterior head of a delicate curette, to facilitate reduction [23].

Extra-articular reduction methods have also been employed by other surgeons, including the use of reduction clamps and Kirschner wires. Jennings et al. described the use of a reduction clamp in a Tillaux fracture case, with the lateral jaw of the clamp placed on the distal tibial epiphysis, medial to the fibula, and tightened to affect the reduction [22]. Similarly, McGillion et al. reported the use of bone reduction clamps applied either over the skin or through small stab incisions to reduce and stabilize the fracture fragments in a triplane fracture [24]. In another case, Thaunat et al. used a Kirschner wire fixed to the fracture fragment, which acted as a joystick to manipulate the fragment while the distal tibial articular surface was arthroscopically controlled to achieve anatomical reduction before advancing the Kirschner wire for temporary fixation [25].

The arthroscopic reduction technique described in this study employs standard tools readily available in operating theaters, making it highly reproducible. The technique involves the use of an accessory portal exclusively for the placement of a bone reduction clamp to accurately reduce and stabilize the fracture fragments before fixation. The additional portal provides enhanced access and maneuverability for fracture reduction, without interfering with the primary anteromedial and anterolateral portals, which remain available for any intra-articular procedures that may be necessary. Notably, there is a scarcity of reports describing arthroscopic reduction techniques that incorporate an additional portal for improved angles of movement, which enhances the precision of reduction. Most published methods typically utilize a maximum of two portals: one for visualization and the other for reduction work.

In terms of healing time, the patients in this study achieved full radiological healing in an average of 45 ± 6 days, significantly faster than other reported cases using alternative arthroscopic reduction techniques (Table 2). For instance, Feng et al. evaluated 19 patients with radiological union occurring at an average of 23.5 ± 6.4 weeks postoperatively [21]. In a case reported by Panagopoulos et al., where a drill sleeve through the anterolateral portal was used for reduction and temporary fixation with a 2.0 Kirschner wire, healing took 12 weeks [17]. Similarly, a case by Ogawa and Shimizu required nine months for bone union, with the epiphysis almost closed [26].

The patients in this study also achieved full weight-bearing ambulation after an average of six weeks (57 ± 7 days), which is consistent with or even superior to other methods, which typically require six weeks or more for weight-bearing progression [16,21]. Some reported cases have still not allowed full weight-bearing after six weeks [18,25].

At the six-month follow-up, patients in this study showed excellent functional outcomes that were superior to those reported for other arthroscopic ankle fracture fixation methods, with average AAOS Foot and Ankle Outcome Questionnaire scores of 93 ± 3 and FAOS scores of 92 ± 5 (Table 1) [11,12,27]. McDonald et al. reported in one of their study groups of 22 patients that underwent ankle arthroscopy, the mean AAOS score of 89.6 ± 7.9, with a mean follow-up time of 20.9 ± 8.0 months [17,28]. This underscores the efficacy of our technique not only in terms of healing time but also in restoring functional status and quality of life.

Arthroscopic fixation of transitional ankle fractures presents several technical challenges. The procedure requires high surgical precision, as reduction and rigid fixation must be achieved through limited incisions and within the constrained capsular space, conditions that are especially demanding in ankle joints. One of the primary intraoperative priorities is to avoid penetrating the articular surface and to preserve joint congruity during screw placement. As highlighted by Ogawa and Shimizu, while preserving the epiphysis is important, precise anatomical reconstruction of the articular surface may at times necessitate crossing into the epiphyseal region, depending on the patient's remaining growth potential and the proportion of the physis involved [26].

This study also has several limitations. Arthroscopic management of transitional fractures requires a steep learning curve and can only be performed by surgeons experienced in pediatric arthroscopy. The small sample size and lack of a control group in this technical report limit the generalizability of the findings. Furthermore, the study reports only short-term outcomes, which may not fully capture the long-term efficacy or potential complications of the technique.

To strengthen the evidence base, future studies should include a larger cohort of patients and assess long-term outcomes following arthroscopic fixation of transitional ankle fractures. Comparative studies that evaluate this technique against conventional open or percutaneous methods are essential to determine its relative advantages and limitations. Additionally, standardizing training protocols and developing simulation-based learning tools may help reduce the learning curve and make the technique more accessible to a broader group of orthopedic surgeons.

## Conclusions

In conclusion, the arthroscopically assisted fixation technique described in this study for Tillaux and two-part triplane fractures has yielded outstanding clinical outcomes, demonstrating excellent healing and functional recovery. By providing a safer and more controlled approach to achieving precise fracture reduction, this technique improves surgical efficiency while maintaining a smooth workflow. Additionally, its reproducibility and use of standard surgical instruments make it a practical and accessible choice for widespread use in diverse clinical settings.
